# Assessing and categorizing health-related quality of life outcomes in children and youth with acquired brain injury in outpatient rehabilitation

**DOI:** 10.1177/18758894261460617

**Published:** 2026-06-16

**Authors:** Florian Allonsius, Celine de Mooij, Arend de Kloet, Frederike van Markus-Doornbosch, Menno van der Holst

**Affiliations:** 1 Dept. Innovation, Quality & Research, 4501 Basalt Rehabilitation, Leiden and The Hague, The Netherlands; 2 Dept. Orthopedics, Rehabilitation and Physical Therapy, Leiden University Medical Center, Leiden, The Netherlands

**Keywords:** acquired brain injury, pediatrics, rehabilitation, quality of life

## Abstract

**Introduction:**

Young patients with acquired brain injury (ABI) often report diminished health-related quality of life (HRQoL) compared to healthy peers. Yet, this has not been investigated in a large multicenter outpatient-rehabilitation based cohort. Furthermore, a clear way to categorize HRQoL to better interpret scores is lacking, though this could be valuable for clinical use (i.e., for comparison with other populations).

**Methods:**

This cross-sectional study used the PedsQL™ Generic Core Scales 4.0 questionnaire with 23 items to assess patients’ HRQoL, where lower scores mean a more diminished HRQoL. Children (8–12 years), adolescents (13–17 years), and young adults (18–25 years) with ABI, referred to a Dutch rehabilitation center, were included. Patient characteristics were analyzed using descriptive statistics and mean (standard deviation [SD]). HRQoL scores were calculated per age group and subsequently categorized using reference data(means, SDs) from healthy peers. HRQoL was categorized as 1 (‘better HRQoL than healthy peers’, > + 1SD), 2 (‘comparable HRQoL’, −1SD to +1SD), 3 (‘moderately diminished HRQoL’, −1SD to −2SD), or 4 (‘severely diminished HRQoL’, < −2SD).

**Results:**

Four hundred twenty-six young patients (aged 8–25 years) with ABI participated in the study; 233 were female (54.7%), and 334 had a diagnosis of traumatic brain injury (78.4%). Children, adolescents, and young adults had mean (SD) HRQoL total scores below age-specific Dutch healthy peer normative means (62.98 [14.39], 62.86 [16.82], and 59.34 [18.46], respectively). Based on the SD-based categorization using healthy peer reference data, 59% of children (55/93), 52% of adolescents (147/282), and 61% of young adults (31/51) were categorized in Category 4 (‘severely diminished HRQoL’ < −2SD relative to age-specific Dutch healthy peer norms).

**Conclusion:**

Measuring and categorizing HRQoL in outpatient rehabilitation may facilitate clinically meaningful interpretation of PedsQL scores by benchmarking to healthy peers, supporting shared decision-making at referral in young patients with ABI.

## Introduction

Acquired brain injury (ABI) includes any damage sustained to the brain after birth that is not caused by a congenital or perinatal condition.^1,2^ ABI can be categorized into traumatic brain injury (TBI), due to external causes such as traffic and sport incidents, and non-traumatic brain injury (nTBI), due to internal causes such as stroke and brain tumors.^1,3,4^ In the Netherlands, the estimated annual incidence of TBI and nTBI among those under the age of 25 is 586 and 191 per 100,000, respectively.^5^ Due to natural adaptation of the brain, approximately 70% of these young patients recover within six months after the injury.^5^ However, an estimated 30% of young patients continue to report persistent problems beyond six months; for this subgroup, multidisciplinary outpatient rehabilitation may be indicated.^2,6^

Studies in pediatric and young adult ABI cohorts report persistent problems across multiple domains of functioning as described by the International Classification of Functioning, Disability and Health (ICF) (e.g., body functions, activities, and participation), which can adversely affect health-related quality of life (HRQoL).^1,5–10^ HRQoL is described as a multidimensional concept, comprised of physical, functional, emotional, and social wellbeing domains which can be affected by perceived health, illness, and injury.^11–13^ In outpatient rehabilitation, assessing HRQoL during the first specialist consultation following referral can help identify patient-reported difficulties and support clinical and shared decision-making regarding rehabilitation goals.^14–20^ When HRQoL is assessed repeatedly during care, it may also support monitoring of change over time.

To assess HRQoL, patient- or parent-reported outcome measures (PROMs) are often used.^21^ Several HRQoL PROMs are available, including the Quality of Life after Brain Injury (QOLIBRI),^22^ the Short Form Health Survey (SF-36),^23,24^ Kidscreen-27,^25^ and KINDL-R.^26^ However, the QOLIBRI and SF-36 are designed for adults, whereas Kidscreen-27 and KINDL-R are validated only up to 18 years, limiting their use across the transition from adolescence to young adulthood.^22,27–29^ Additionally, the World Health Organization (WHO) states that HRQoL instruments should be multidimensional, including at minimum physical, social and psychological (including cognitive and emotional) dimensions.^30^ The Pediatric Quality of Life Inventory™ Generic Core Scales 4.0 (PedsQL GCS)^31^ is widely used in youth (5–30 years), including in ABI populations; spans the child-to-adult transition period; and covers physical, emotional, social and school/work functioning.^10,31–37^ The PedsQL GCS has adequate validity, feasibility and reliability and is available in Dutch.^31–33,38^ It provides a 0–100 score, with lower scores indicating more diminished HRQoL.^31^ While this scale is intuitive, clinicians and families often benefit from normative benchmarks or cut-offs to interpret whether a given score reflects clinically relevant impairment relative to healthy peers. These benchmarks or cut-offs can also support communication and clinical decision-making. Moreover, when HRQoL is assessed repeatedly during care, a norm-referenced categorization may support interpretation of changes over time, particularly since minimal clinically important differences (MCIDs) for outpatient ABI populations are not well established. Using cut-off points alongside the conventional 0–100 scores may therefore facilitate an additional clinically meaningful interpretation of HRQoL outcomes.

Previous studies using the PedsQL GCS have suggested that scores ≥1 standard deviation (SD) below the mean of a healthy population may indicate ‘at-risk’ HRQoL.^31,39^ Moreover, studies using PedsQL instruments in pediatric populations (e.g., the PedsQL Multidimensional Fatigue Scale) have categorized scores ≥2 SD below healthy normative means as reflecting ‘severely’ diminished scores.^10,40^ Building on these established, distribution-based thresholds, the same SD-based approach was applied to HRQoL to create a four-level categorization that benchmarks patient scores against healthy peers.

Cut-off points based on score distributions in healthy populations may provide a useful framework for categorizing HRQoL scores. This approach enables comparison with healthy peers and may facilitate the clinical interpretation of symptoms throughout the rehabilitation process. No such studies have been conducted to date. Therefore, this study aimed to assess the HRQoL of young patients with ABI (8–25 years) at referral to outpatient rehabilitation, compare their HRQoL with that of healthy Dutch age-matched peers, and develop an HRQoL classification system to provide patients, parents, and healthcare professionals with a quick interpretation of HRQoL scores in clinical practice.

## Methods

### Design and setting

This cross-sectional study was part of a larger multicenter research project on HRQoL, participation, fatigue, and family impact using a questionnaire. The larger study included Dutch children, adolescents, and young adults, aged four to 25 years, and their families. The project was conducted in 14 rehabilitation centers in the Netherlands between 2015 and 2023. The medical ethics committee of the Leiden University Medical Center provided an exemption from full medical ethical review for this project (P15.165, and P15.165-addendum-1.0). Additionally, approval from all participating centers was acquired. For the current study, only patient-reported data on HRQoL at the time of referral were used. To report study outcomes, the Strengthening the Reporting of Observational studies in Epidemiology (STROBE) guidelines were used.^41^

### Participants

Children, adolescents, and young adults aged 8–25 years with ABI who were referred to one of the participating rehabilitation centers were eligible to participate in this study. For those under the age of eight years, parents completed a questionnaire; thus, this age group was not included in the current study. Patients under the age of 16 years had to receive permission from their parents to participate according to the Dutch law of healthcare decision-making. Participants completed a questionnaire containing a set of PROMs, including the PedsQL GCS.^32^ Participants were excluded if they were insufficiently proficient in the Dutch language or if their questionnaire was incomplete, such that PedsQL GCS scores could not be calculated. Administering the questionnaire at referral to rehabilitation was part of regular care, and therefore, informed consent was not required.

### Procedure

Data collection:

The questionnaire involved a series of outcome measures (including HRQoL), administered digitally or on paper, at admission to one of the participating rehabilitation centers. In the digital version, instruments were presented sequentially, and participants could discontinue at any point; the HRQoL measure (PedsQL GCS) was included toward the end of the questionnaire, leading to partial completion. PedsQL GCS total and domain scores were calculated according to the instrument scoring guidelines and were computed only when sufficient items were completed. Questionnaires for which PedsQL GCS scores could not be calculated were excluded. Participants completed the questionnaire either at home or at the outpatient clinic, using digital or paper formats. Unique links to the digital questionnaire were emailed to participants by medical secretaries from the centers. Paper-based questionnaires were manually entered into the digital system by the data manager after completion. All data were subsequently anonymized and securely stored in a central database at Basalt Rehabilitation Center in the Hague, Netherlands. For this study, only HRQoL data collected at admission were analyzed.

Demographic- and injury-related characteristics:

Patient demographic- and injury-related characteristics were extracted from medical records by medical secretaries at the rehabilitation centers. Demographic characteristics included sex (male/female) and age (in years, on the date of first visit). For injury-related characteristics, the cause of ABI (TBI/nTBI) was reported. When available, the Glasgow Coma Scale (GCS) score at hospital admission was used to classify TBI severity as either mild (GCS ≥13) or moderate/severe (GCS <13).^42^ If there was no hospital admission or GCS reported, patients with TBI with no history of loss of consciousness were equally considered as ‘mild’. Causes of nTBI were further categorized as stroke, tumor, meningitis/encephalitis, or hypoxia/intoxication. If the cause of nTBI was not reported, it was categorized as unknown. Time since injury/onset at referral was not consistently available across centers and, therefore, not included.

Outcome measure:

The Dutch version of the PedsQL GCS was used to assess HRQoL. This PROM has demonstrated adequate validity, feasibility, and reliability in both healthy populations and populations with chronic health conditions, including TBI.^16,32,34,38,43,44^ The 23-item PedsQL GCS has age-specific versions and yields a total score as well as four dimension scores. The dimensions are Physical Functioning, eight items (e.g., ‘It is hard for me to run’); Emotional Functioning, five items (e.g., ‘I feel sad or blue’); Social Functioning, five items (e.g., ‘Other kids don’t want to be my friend’); and School/Work Functioning, five items (e.g., ‘I have trouble keeping up with my (school)work’). All items are scored on a five-point Likert scale from 0 = never to 4 = almost always. After completion of the questionnaire, all items are linearly transformed to a scale from zero to 100 (0 = 100, 1 = 75, 2 = 50, 3 = 25, 4 = 0). To create the total and dimension scores, the sum of all items for the total score and the sum of all items within a domain are divided by the number of items answered in the specific domain. Lower scores indicate a more diminished HRQoL.

Categorization of the PedsQL GCS:

Data from previously published studies regarding HRQoL in healthy Dutch children, adolescents, and young adults, collected with the PedsQL GCS, were used to categorize and interpret the scores of young patients with ABI.^43,44^ Engelen et al. included 496 healthy, school-going children in age groups 5–7 years (parent-/proxy-reported), 8–12 years (self-reported) and 13–18 years (self-reported).^43^ Limperg et al. included 310 healthy young adults between 18–25 years.^44^ A four-point categorization of the PedsQL GCS is proposed based on these published aggregated results (mean and SD) of the reference data of healthy peers.^43,44^

SD-based cut-offs were chosen because the available Dutch normative reference data are reported as age-specific means and SDs,^43,44^ enabling a transparent and reproducible categorization without requiring additional conversion tables. This approach is consistent with prior PedsQL work suggesting that scores ≥1 SD below normative means indicate ‘at-risk’ outcomes and that ≥2 SD below norms reflects a more marked deviation.^31,39^ Percentile and T-score approaches are mathematically related to SD-based thresholds. However, these could not be directly derived from the published aggregated Dutch norms because percentile ranks/T-score conversions were not provided.

Cut-off points for each age group were determined using the means and SDs of the total and dimension scores of the reference data. Thereafter, the total and dimension scores of young patients with ABI were compared to the scores of healthy peers. Categorization was based on how far each participant's PedsQL GCS score deviated from the age-specific normative mean, expressed in SD units (z-score distance).^43,44^

**Figure 1.**
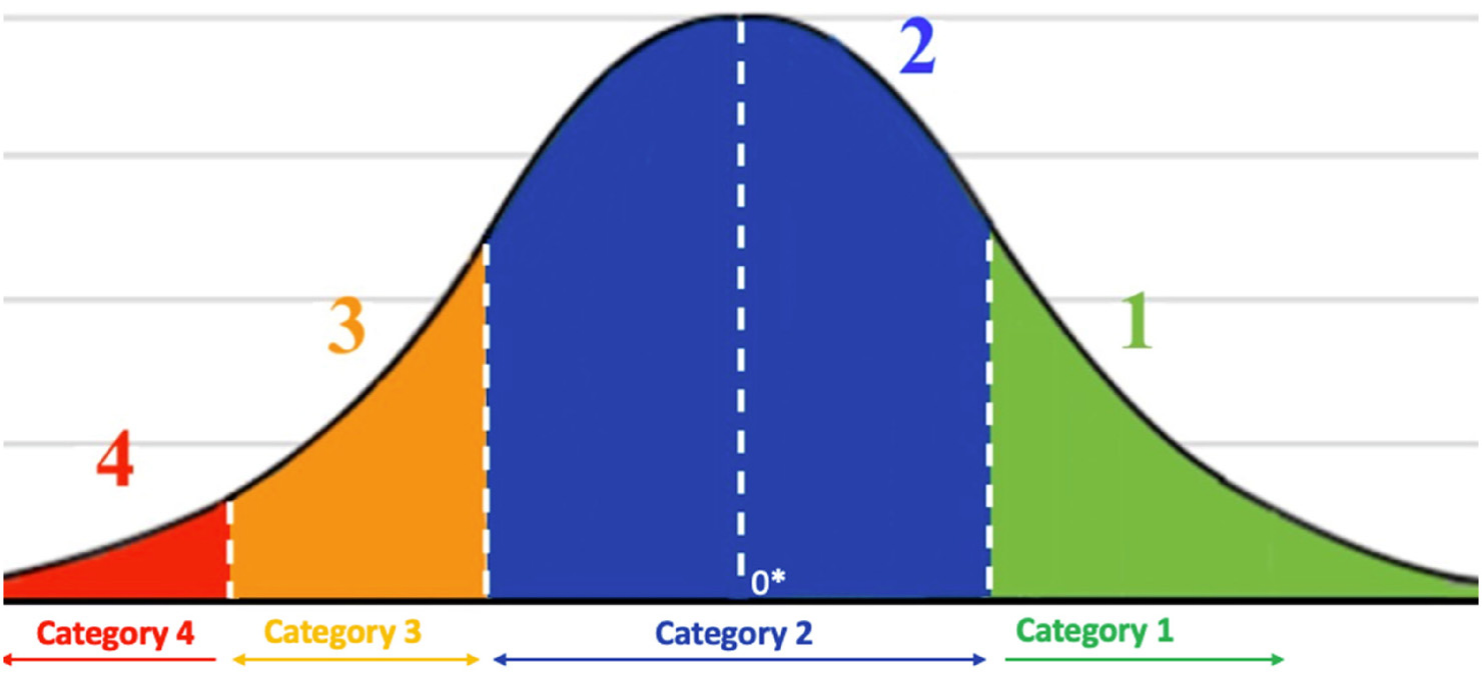
Four-point classification in a normal distribution curve.

The four-point categorization is as follows (Figure 1):

- **Category 1:** Score > + 1 SD: Better HRQoL compared to healthy peers
- **Category 2:** Score between +1 SD and −1 SD: Comparable to healthy peers
- **Category 3:** Score between −1 SD and −2 SD: Moderately diminished compared to healthy peers
- **Category 4:** Score < −2 SD: Severely diminished compared to healthy peers

0*: equal to mean score of reference data.

### Data analyses

All demographic- and injury-related characteristics were analyzed using descriptive statistics. Patients were divided into age groups: 8–12, 13–17, and 18–25 years. The PedsQL GCS outcomes of young patients with ABI were analyzed using descriptive statistics and expressed as means (SDs) or medians (interquartile ranges) based on their distribution. The Kolmogorov–Smirnov test was used to assess normality of the HRQoL score distributions. Thereafter, the PedsQL GCS outcomes (continuous variables) from young patients with ABI were benchmarked against the aggregated data (means and SDs) of healthy peers.^43,44^

Because the Dutch reference data were available only as published aggregated means and SDs,^43,44^ conventional two-sample statistical tests could not be performed. Therefore, differences between the mean HRQoL scores of young patients with ABI and the reference means were expressed in SD units using an aggregated Z-score approach per age group and per PedsQL dimension. Accordingly, comparisons are presented descriptively as SD-based deviations from healthy peer norms using the following formula:

$$Z=\;\frac{\left(X-\;\mu \right)}{\sigma }$$

where “X” (the mean HRQoL score of young patients with ABI) minus “µ” (the mean HRQoL score of healthy Dutch peers) is divided by “σ” (the SD of the mean HRQoL score in healthy Dutch peers). This calculation was performed per age group and per dimension score. Z-values were used to quantify the extent of deviation in SD units and to support the SD-based four-level categorization.

All statistical analyses were performed by using the Statistical Package for the Social Sciences version 29.0 (IBM SPSS Statistics for Mac, Armonk, NY: IBM Corp).

## Results

**Figure 2.**
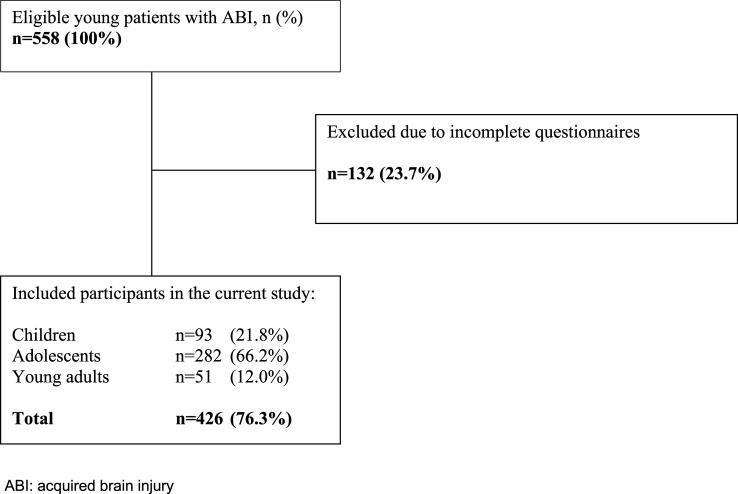
Flowchart of participant inclusion.

Data from 426 participants were used in this current study (Figure 2). The total cohort consisted of 93 (21.8%) children (8–12 years), 282 (66.2%) adolescents (13–17 years) and 51 (12.0%) young adults (18–25 years). More than half of the participants were female (*n* = 233, 54.7%). Of all participants, 334 (78.4%) had a TBI, of which *n* = 274 (82.0%) were classified as ‘mild’. For 92 participants (21.6%), injury was classified as nTBI with stroke (*n* = 21, 22.8%) and tumor (*n* = 37, 40.2%) as the most frequently reported causes. All patient characteristics are presented in Table 1.

ABI: acquired brain injury.

**Table 1.** Demographic- and injury-related characteristics of young patients with ABI per age group.

	Total cohort n = 426		Children* n = 93 (21.8%)		Adolescents** n = 282 (66.2%)		Young adults*** n = 51 (12%)	
**Age**								
Years, Mean (SD)	14.61	(2.91)	10.48	(1.27)	15.15	(1.39)	19.20	(1.86)
**Gender**								
Female, n (%)	233	(54.7%)	48	(51.6%)	154	(54.6%)	31	(60.8%)
**TBI, n (%)**	334	(78.4%)	63	(67.7%)	229	(81.2%)	42	(82.4%)
**Severity levels, n (%)**	274	(82.0%)	51	(81.0%)	187	(81.7%)	36	(85.7%)
Mild (GCS ≥13)								
Moderate/severe (GCS <13)	36	(10.8%)	3	(4.8%)	28	(12.2%)	5	(11.9%)
Unknown****	24	(7.2%)	9	(14.3%)	14	(6.1%)	1	(2.4%)
**nTBI, n (%)**	92	(21.6%)	30	(32.3%)	53	(18.8%)	9	(17.6%)
**Causes, n (%)**								
Stroke	21	(22.8%)	3	(10.0%)	14	(26.4%)	4	(44.4%)
Tumor	37	(40.2%)	13	(43.3%)	21	(39.6%)	3	(33.3%)
Meningitis/encephalitis	13	(14.1%)	6	(20.0%)	6	(11.3%)	1	(11.1%)
Hypoxia/intoxication	4	(4.3%)	1	(3.3%)	2	(3.8%)	1	(11.1%)
Unknown	17	(18.5%)	7	(23.3%)	10	(18.9%)	0	(0.0%)

*8–12 years, **13–17 years, ***18–25 years, ****If the GCS was unknown/not applicable for these patients, and if they had no history of consciousness loss at onset, the severity was considered mild.

ABI: acquired brain injury; TBI: traumatic brain injury; nTBI: non-traumatic brain injury; GCS: Glasgow Coma Scale.

### HRQoL in young patients with ABI

The means and SDs of the total and dimension scores on the PedsQL GSC across all age groups are presented in Table 2. The mean (SD) total score was 62.98 (14.39) in children, 62.86 (16.82) in adolescents, and 59.34 (18.46) in young adults. In all age groups, the dimension ‘School/Work Functioning’ was scored lowest, and the dimension ‘Social Functioning’ was scored highest.

**Table 2.** Patient-reported HRQoL in children, adolescents and young adults with ABI benchmarked against healthy Dutch peers.

	**Patients with ABI**		**Healthy peers**		**Mean difference**	z-value (SD-based deviation)*
**Total cohort 8–25 years**	**n = 426**					
	**mean**	**(SD)**				
**HRQoL total score^#^**	**62.52**	**(16.54)**				
Physical functioning	65.43	(22.87)				
Emotional functioning	62.19	(22.68)				
Social functioning	73.70	(18.56)				
School/work functioning	47.92	(21.44)				
**Children 8-12 years**	**n = 93**		**n = 219^a^**			
	**mean**	**(SD)**	**mean**	**(SD)**		
**HRQoL total score^#^**	**62.98**	**(14.39)**	**82.11**	**(8.87)**	**19.13**	**2.2**
Physical functioning	65.63	(21.19)	80.63	(10.31)	15.00	**−**1.5
Emotional functioning	61.13	(22.22)	77.05	(13.66)	15.92	**−**1.2
Social functioning	71.51	(15.77)	86.14	(12.30)	14.63	**−**1.2
School/work functioning	52.74	(18.45)	78.70	(12.00)	26.23	**−**2.2
**Adolescents 13-17 years**	**n = 282**		**n = 185^a^**			
	**mean**	**(SD)**	**mean**	**(SD)**		
**HRQoL total score^#^**	**62.86**	**(16.82)**	**82.24**	**(9.15)**	**19.38**	**−2.1**
Physical functioning	66.03	(22.67)	80.23	(10.18)	14.20	**−**1.4
Emotional functioning	63.14	(22.65)	76.70	(15.20)	13.56	**−**0.9
Social functioning	74.57	(19.30)	89.38	(11.56)	14.80	**−**1.3
School/work functioning	47.36	(21.94)	74.59	(13.16)	27.23	**−**2.1
**Young Adults 18-25 years**	**n = 51**		**n = 310^a^**			
	**mean**	**(SD)**	**mean**	**(SD)**		
**HRQoL total score^#^**	**59.34**	**(18.46)**	**85.90**	**(11.18)**	**26.56**	**−2.4**
Physical functioning	61.77	(26.73)	90.20	(12.45)	28.43	**−**2.3
Emotional functioning	58.92	(23.76)	78.42	(17.68)	19.50	**−**1.1
Social functioning	72.84	(19.03)	88.44	(13.66)	15.60	**−**1.1
School/work functioning	42.26	(22.28)	83.95	(14.26)	41.69	**−**2.9

^a^Dutch reference data from healthy peers (self-reported): ages 8–12 (children), 13–17 (adolescents), ≥18 (young adults) years old.

^#^HRQoL, measured with the PedsQL Generic Core Scales 4.0, 0–100, with lower scores indicating more diminished HRQoL.

*Z indicates SD-based deviation from healthy peer norms: Z = the mean HRQoL score of young patients with ABI, minus the mean HRQoL score of healthy Dutch peers, divided by the SD of the mean HRQoL score in healthy Dutch peers.

HRQoL: health-related quality of life; ABI: acquired brain injury.

### HRQoL benchmarked to healthy peer norms

Table 2 presents descriptive benchmarking of HRQoL scores in young patients with ABI against age-specific Dutch healthy peer normative means and SDs. Mean HRQoL total scores in young patients with ABI were 19.13, 19.38, and 26.56 points below the normative means for children, adolescents, and young adults, respectively (Table 2). Across domains, the largest deviations from norms were observed in ‘School/Work Functioning’ (26.23, 27.23, and 41.69 points below norms for children, adolescents, and young adults, respectively), as well as in ‘Physical Functioning’ for young adults (28.43 points below norms).

### Four-point categorization of HRQoL in young patients with ABI based on data from healthy peers

**Figure 3.**
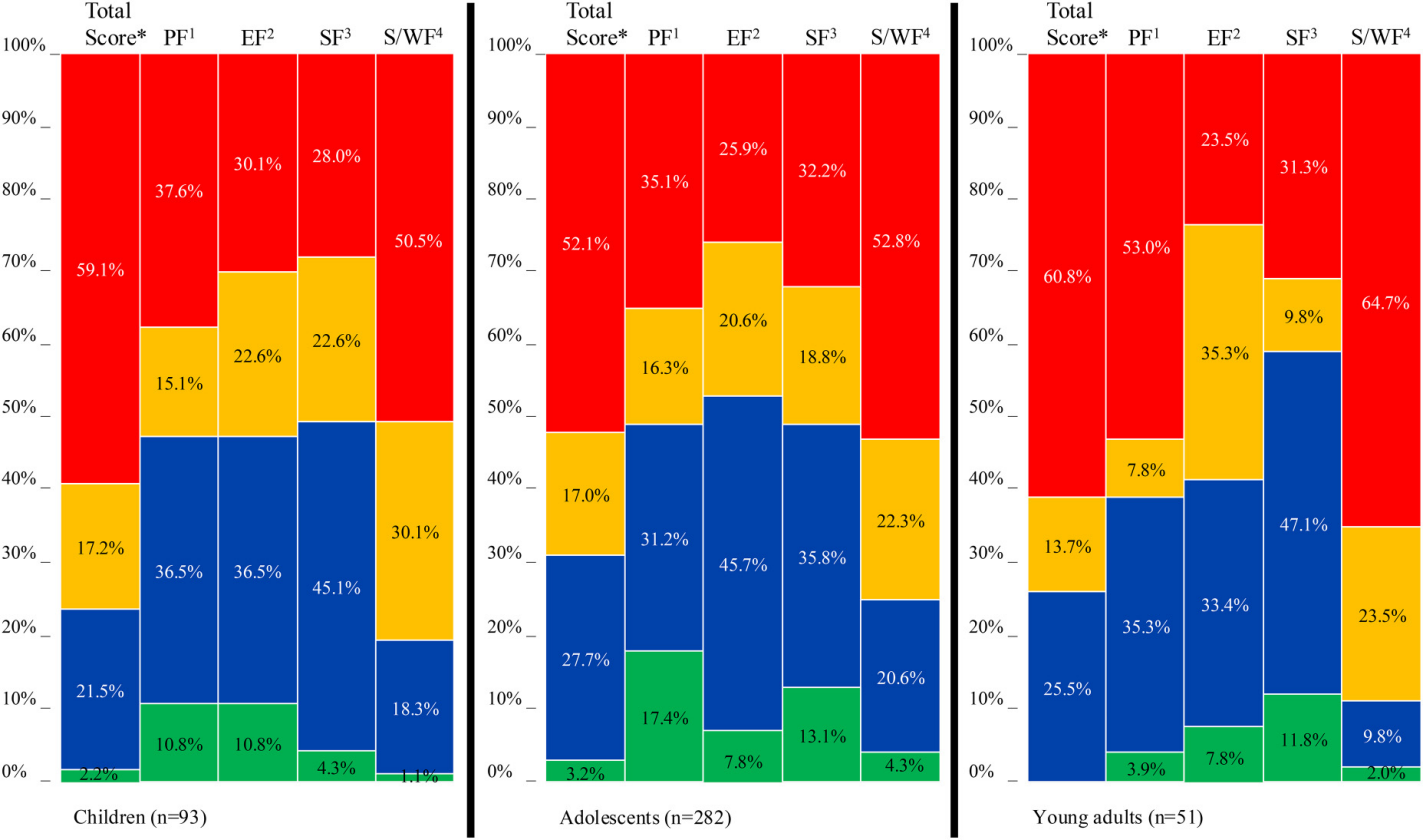
Percentages of children/adolescents/young adults with ABI per HRQoL category on the PedsQL generic core scales 4.0

The ranges of the categorization, number (n), and percentages of patients per category for all total and domain scores are presented in Table 3. Figure 3 presents the percentages for each category. The majority of young patients with ABI had total scores that fell into Category 4, ‘severely diminished HRQoL compared to healthy peers’ (59.1% of the children, 52.1% of adolescents and 60.8% of young adults). In the dimension ‘School/Work Functioning,’ the majority of patients had scores in the category ‘severely diminished compared to healthy peers’ as well (50.5% of children, 52.8% of adolescents, and 64.7% of young adults).

**Category 1:** > + 1 SD: Better HRQoL compared to healthy peers. **Category 2:** +1SD and −1SD: Comparable to healthy peers. **Category 3:** −1SD and −2SD: Moderately diminished compared to healthy peers. **Category 4:** < −2SD: Severely diminished HRQoL compared to healthy peers. *Numbers and percentages of participants per category. ABI: acquired brain injury; HRQoL: health-related quality of life; PF: physical functioning; EF: emotional functioning; SF: social functioning; S/WF: school/work functioning.

**Table 3.** Four-point classification based on total and domain PedsQL generic core scales 4.0 scores and sds from Dutch reference data.

	**Total score mean (SD)**		n (%)*	**PF mean (SD)**	n (%)*	**EF mean (SD)**	n (%)*	**SF mean (SD)**	n (%)*	**S/WF mean (SD)**	n (%)*
**Children** **8–12 years** (n = 93)	82.11 (8.87)			80.63 (10.31)		77.05 (13.66)		86.14(12.30)		78.70 (12.00)	
	** Category 1**	100–90.98	2 (2.2)	100–90.94	10 (10.8)	100–90.71	10 (10.8)	100–98.44	4 (4.3)	100–90.70	1 (1.1)
	**Category 2**	90.98–73.24	20 (21.5)	90.94–70.32	34 (36.5)	90.71–63.39	34 (36.5)	98.44–73.84	42 (45.1)	90.70–66.70	17 (18.3)
	**category 3**	73.24–64.37	16 (17.2)	70.32–60.01	14 (15.1)	63.39–49.73	21 (22.6)	73.84–61.54	21 (22.6)	66.70–54.70	28 (30.1)
	**Category 4**	< 64.37	55 (59.1)	< 60.01	35 (37.6)	< 49.73	28 (30.1)	< 61.54	26 (28.0)	< 54.70	47 (50.5)
**Adolescents** **13–17 years** (n = 282)	82.24 (9.15)			80.23 (10.18)		76.70 (15.20)		89.38 (11.56)		74.59 (13.16)	
	**Category 1**	100 ─ 91.39	9 (3.2)	100 ─ 90.41	49 (17.4)	100 ─ 91.90	22 (7.8)	100 ─ 100	37 (13.1)	100–87.75	12 (4.3)
	**Category 2**	91.39 ─ 73.09	78 (27.7)	90.41 ─ 70.05	88 (31.2)	91.90 ─ 61.50	129 (45.7)	100 ─ 77.82	101 (35.8)	87.75 ─ 61.43	58 (20.6)
	**Category 3**	73.09 ─ 63.94	48 (17.0)	70.05 ─ 59.87	46 (16.3)	61.50 ─ 46.30	58 (20.6)	77.82 ─ 66.26	53 (18.8)	61.43 ─ 48.30	63 (22.3)
	**Category 4**	< 63.94	147 (52.1)	< 59.87	99 (35.1)	< 46.30	73 (25.9)	< 66.26	91 (32.3)	< 48.30	149 (52.8)
**Young adults** **18–25 years** (n = 51)	85.90 (11.18)			90.20 (12.45)		78.42 (17.68)		88.44 (13.66)		83.95 (14.26)	
	**Category 1**	100 ─ 97.08	0 (0.0)	100 ─ 100	2 (3.9)	100 ─ 96.10	4 (7.8)	100 ─ 100	6 (11.8	100–98.21	1 (2.0)
	**Category 2**	97.08 ─ 74.72	13 (25.5)	100 ─ 77.75	18 (35.3)	96.10 ─ 60.74	17 (33.4)	100 ─ 74.78	24 (47.1)	98.21 ─ 69.69	5 (9.8)
	**Category 3**	74.72 ─ 63.54	7 (13.7)	77.75 ─ 65.30	4 (7.8)	60.74 ─ 43.06	18 (35.3)	74.78 ─ 61.12	5 (9.8)	69.69 ─ 55.43	12 (23.5)
	**Category 4**	< 63.54	31 (60.8)	< 65.30	27 (53.0)	< 43.06	12 (23.5)	< 61.12	16 (31.3)	< 55.43	33 (64.7)

PF = Physical functioning; EF = Emotional functioning; SF = Social functioning; S/WF = School/work functioning.

*Numbers and percentages of participants per category.

## Discussion

This cross-sectional study assessed HRQoL using the PedsQL GSC in young patients with ABI (aged 8–25 years) who were referred to outpatient rehabilitation. Across all age groups (8–12, 13–17, and 18–25 years), mean PedsQL GSC total scores deviated negatively from age-specific Dutch healthy peer normative means. A four-point HRQoL categorization for young patients with ABI was proposed using cut-off points based on scores and SDs of healthy age-matched peers. This categorization showed that the majority of young patients with ABI across all age groups were categorized as having a ‘severely diminished HRQoL’ according to the SD-based, norm-referenced categorization (Category 4: < −2 SD compared to age-specific Dutch healthy peer data).

### HRQoL in children, adolescents, and young adults with ABI in the rehabilitation setting

Regarding the total population of young patients in this study, the most diminished HRQoL scores were found in the ‘School/Work Functioning’ dimension of the PedsQL GSC. When looking at age subgroups, more diminished HRQoL scores were found in young adults compared to children and adolescents.

Both of these results were generally in line with a previous study that had a much smaller rehabilitation-based cohort.^45^ Low HRQoL scores also be influenced by fatigue and participation restrictions, since there is a known longitudinal correlation between HRQoL, fatigue, and participation in populations of young patients with ABI in the rehabilitation setting.^46^

The overall low HRQoL scores in this study warrant extra attention at admission and during outpatient rehabilitation treatment in the Netherlands.

### HRQoL in young patients with ABI compared to healthy peers

The HRQoL scores were, on average, approximately 20 points below the age-specific Dutch healthy peer normative means. These results were generally in line with scores found in a previous study showing lower HRQoL scores in hospitalized TBI patients (0–17 years) compared to a control group.^47^ Addressing diminished HRQoL early in rehabilitation could improve long-term outcomes through targeted interventions based on specific HRQoL dimensions. However, further research is needed to assess long-term HRQoL outcomes and evaluate these interventions.

### The young adult age group

When focusing specifically on the young adult age group (aged 18–25 years), a larger mean difference compared to healthy peers was found on all dimensions of HRQoL. This age group showed the lowest scores in the dimension ‘School/Work Functioning.’ This could be attributed to the increasing demands and responsibilities of education and/or work among young adults with ABI.^48,49^ Additionally, this life phase is often characterized by increasing independence and psychosocial role expectations (e.g., balancing study/work with social participation), which may further contribute to lower perceived HRQoL in young adults with ABI. Ensuring a better transition for young adults with ABI into work-related roles^50^ and assessing HRQoL before and after treatment could aid in supporting this specific age group. However, because the young adult subgroup was relatively small (*n* = 51), the observed differences should be interpreted cautiously and warrant confirmation in larger young adult samples.

### Four-point categorization of HRQoL of young patients with ABI based on reference data

To the authors’ knowledge, this is the first study categorize HRQoL scores to compare young patients with ABI with healthy peers. Conceptually, this four-level categorization is a discretized representation of z-score (SD) ranges relative to age-specific Dutch norms and is intended as an interpretive aid alongside continuous scores and z-distance reporting. In this study, the majority of young patients with ABI were categorized as ‘severely diminished compared to healthy peers’ according to the SD-based, norm-referenced categorization.

Notably, Category 4 reflects scores < −2 SD below age-specific Dutch healthy peer norms, a threshold that would be expected in only a small minority of individuals in a normally distributed reference population (approximately 2–3%). The substantially higher proportion observed in this study should therefore be interpreted in the context of this clinically referred outpatient rehabilitation cohort. Participants were referred because of persistent, multidimensional difficulties requiring multidisciplinary care; consequently, referral/selection processes may have enriched the sample with more symptomatic individuals. Accordingly, these category proportions should not be generalized as prevalence estimates for the broader population of young people with ABI.

Previously, Varni et al. introduced the definition of ‘at risk HRQoL’ as < 1SD below the mean of the norm population). Using this definition, an even larger number of young patients with ABI across all age groups in the current study had ‘at risk HRQoL’.^31^ A four-point categorization with multiple cut-off points can be a promising tool for interpreting and differentiating HRQoL scores. It is expected that in clinical (rehabilitation) practice, next to the conventional 0–100 scores, categorizing HRQoL could provide valuable insight for both the patients and/or their parents and healthcare professionals, allowing for an easy and quick comparison to peers. It may support interpretation and communication at referral/admission. However, clinical utility requires prospective evaluation. Additionally, using HRQoL cut-off categorization has the potential to better evaluate treatment effectiveness instead of just comparing scores over time. For instance, a patient in the ‘severely diminished HRQoL’ category may improve to ‘comparable to healthy peers’ post-treatment. Therefore, future research should focus on longitudinal studies in young patients with ABI in the rehabilitation setting where HRQoL is being assessed and categorized over time.

### Limitations

First, a complete severity classification of TBI could not be displayed, since access was limited to GCS scores for some patients. Not all patients were seen in a hospital, where brain injury severity is normally assessed after ABI onset in the acute phase. Therefore, some patients with TBI were reported with ‘unknown’ severity. However, whether there was a history of consciousness loss at onset was verified by the rehabilitation centers for patients with ‘unknown’ severity levels. None had consciousness loss, and the TBI severity of these patients could be equally considered as ‘mild.’

Secondly, only self-reported Dutch reference data from healthy peers was available,^43,44^ which prevented the inclusion of parent-reported scores in the four-level HRQoL categorization. As a result, the parents’ perspectives were not available in this study.

Thirdly, generalizability may be limited for several reasons. The cohort included a slightly higher proportion of females than males (54.7%). As TBI incidence cohorts are often male-predominant, the sex distribution in this study may limit generalizability to the broader TBI population. The young adult subgroup (18–25 years) was relatively small (n = 51) and may be underrepresented due to practice variation across participating centers in upper age limits (often up to 18 years only), with some young adults being referred to adult services and therefore not captured in this cohort; this may limit the generalizability of age-specific findings for young adults. In addition, some participants were excluded due to incomplete questionnaire completion; in the digital questionnaire battery, the HRQoL measure (PedsQL GCS was positioned toward the end, and participants could discontinue at any time. Because reasons for non-completion and socioeconomic characteristics were not systematically available, potential nonresponse patterns could not be examined, which may further limit generalizability. The time since injury/onset at referral was not consistently available, and associations between time post-injury and HRQoL were therefore unable to be examined. Accordingly, these findings should be interpreted as describing HRQoL at referral/admission among young patients referred to outpatient rehabilitation (i.e., a clinically indicated subgroup), including those with mild TBI. The SD-based category proportions reported in this study should not be interpreted as prevalence estimates for all young individuals with ABI.

Finally, as with every self-reported outcome measure, the results could be influenced by lack of motivation, or moment-bound stress and mood.

## Conclusion

The majority of young patients with ABI referred to outpatient rehabilitation were categorized as having ‘severely diminished HRQoL’ compared to healthy peers according to the SD-based, norm-referenced categorization (Category 4: < −2 SD compared to age-specific Dutch healthy peer norms) at referral. Categorizing HRQoL appears to be promising for use in the outpatient rehabilitation setting as a tool to better target HRQoL scores and provide more insight for patients, parents, and professionals at the start of rehabilitation treatment. When HRQoL is measured repeatedly during care, this categorization may additionally support monitoring change over time and interpreting potential treatment-related improvements; however, this requires longitudinal evaluation.

## Key messages

- Categorizing HRQoL in a four-point cut-off appears to be promising for use in the outpatient rehabilitation setting.
- Young patients with ABI in outpatient rehabilitation show HRQoL scores below age-specific Dutch healthy peer normative means.
- Young adults with ABI are more likely to have more diminished HRQoL-related problems than children and adolescents.
